# Altered aspects of anxiety-related behavior in kisspeptin receptor-deleted male mice

**DOI:** 10.1038/s41598-018-21042-4

**Published:** 2018-02-12

**Authors:** Sarah Delmas, Robert Porteous, Dave H. Bergin, Allan E. Herbison

**Affiliations:** 1 Centre for Neuroendocrinology and Department of Physiology, University of Otago School of Biomedical Medical Sciences, Dunedin, 9054 New Zealand; 2 Department of Anatomy and Centre for Brain Research, University of Otago School of Biomedical Medical Sciences, Dunedin, 9054 New Zealand

## Abstract

The roles of kisspeptin signaling outside the hypothalamus in the brain are unknown. We examined here the impact of *Kiss1r*-deletion on hippocampus-related behaviors of anxiety and spatial learning in adult male mice using two mouse models. In the first, global *Kiss1r*-null and control mice were gonadectomized (GDX KISS1R-KO). In the second, KISS1R signalling was rescued selectively in gonadotropin-releasing hormone neurons to generate *Kiss1r*-null mice with normal testosterone levels (intact KISS1R-KO). Intact KISS1R-KO rescue mice were found to spend twice as much time in the open arms of the elevated plus maze (EPM) compared to controls (*P* < 0.01). GDX KISS1R-KO mice showed a similar but less pronounced trend. No differences were detected between intact KISS1R-KO mice and controls in the open field test (OFT), although a marked reduction in time spent in the centre quadrant was observed for all GDX mice (*P* < 0.001). No effects of KISS1R deletion or gonadectomy were detected in the Morris water maze. These observations demonstrate that KISS1R signalling impacts upon anxiogenic neural circuits operative in the EPM, while gonadal steroids appear important for anxiety behaviour observed in the OFT. The potential anxiogenic role of kisspeptin may need to be considered in the development of kisspeptin analogs for the clinic.

## Introduction

Kisspeptin was identified in 1996 from human melanoma cell lines^1^ and the kisspeptin receptor identified shortly afterwards as G-protein-coupled receptor 54^2–4^, now termed KISS1R^5^. In 2003, the critical importance of kisspeptin for reproduction was established by seminal studies reporting that humans with mutations in KISS1R failed to go through puberty and were infertile^6,7^. Investigations in various animal models over the last decade have now explained those clinical findings by demonstrating that kisspeptin activates KISS1Rs expressed by gonadotropin-releasing hormone (GnRH) neurons to play a central role in the control of fertility^8–10^.

In addition to the GnRH neurons, kisspeptin signaling occurs at cells in several body organs^11,12^ as well as within multiple brain regions outside the hypothalamus^13^. Indeed, kisspeptin-immunoreactive fibers are located throughout the limbic forebrain^14–16^ and cells expressing KISS1R are dispersed across an even wider distribution of the central nervous system^17–19^. In our own mapping studies, we were interested to note that the highest density of cells transcribing *Kiss1r* existed in the dentate gyrus of the hippocampus^18^. Moreover, early electrophysiological studies by Arai and colleagues^20^ reported that kisspeptin selectively modulated excitatory synaptic activity in dentate granule neurons. These data suggested that kisspeptin signaling may have a role in hippocampal functioning.

In the present study, we have examined whether the deletion of *Kiss1r* impacts upon hippocampal-related behavioral tasks such as anxiety and spatial learning^21,22^. *Kiss1r-*null mice fail to go through puberty and remain sexually immature with very low levels of circulating gonadal steroid hormones^23^. This creates an experimental confound as gonadal steroids modulate the activity of many neuronal circuits in the brain, including those within the hippocampus controlling traits such as anxiety^24,25^. One strategy for overcoming this caveat has been to undertake experiments in gonadectomized (GDX) mice so that gonadal steroids are absent in both controls and *Kiss1r*-null mice^26^. However, this results in the investigation of GDX mice rather than normal animals and is, itself, problematic when gonadal steroids may play a role in the parameters being examined. An alternative approach is to use a mouse model in which *Kiss1r* has been deleted from all cells in the body but rescued selectively in the GnRH neurons^27^. This rescue approach results in *Kiss1r*-deleted male mice that have normal levels of circulating testosterone and fertility^27,28^. We have examined here the learning and anxiety traits of *Kiss1r*-null male mice in both the GDX and rescue models to explore whether kisspeptin may have a role in hippocampal function.

## Results

Adult male 129S6 global *Kiss1r*−/− and littermate wild-type *Kiss1r*+/+ mice^7^ (*Kiss1*^rtm1Coll^, provided by Prof. William Colledge, University of Cambridge) were GDX three weeks before beginning experiments when 4–6 months old. Experimental mice were labelled as “GDX KISS1R-KO” and controls as “GDX control”. Adult male *Kiss1r*^−/−^; Tg^+/−^ and littermate Tg^+/−^ mice^27^ were used for experiments at 4–6 months of age. This was a 129P2 global *Kiss1R*-null mouse (*Kiss1*^rtmGstn^) crossed on to a C57BL/6 J line incorporating a transgene (Tg) in which the *Gnrh1* promoter drives the expression of *Kiss1R*^27,29^ (available from AEH). Experimental mice were labelled “intact KISS1R-KO rescue” and controls as “intact controls”.

### Elevated Plus Maze (EPM)

**Figure 1:**
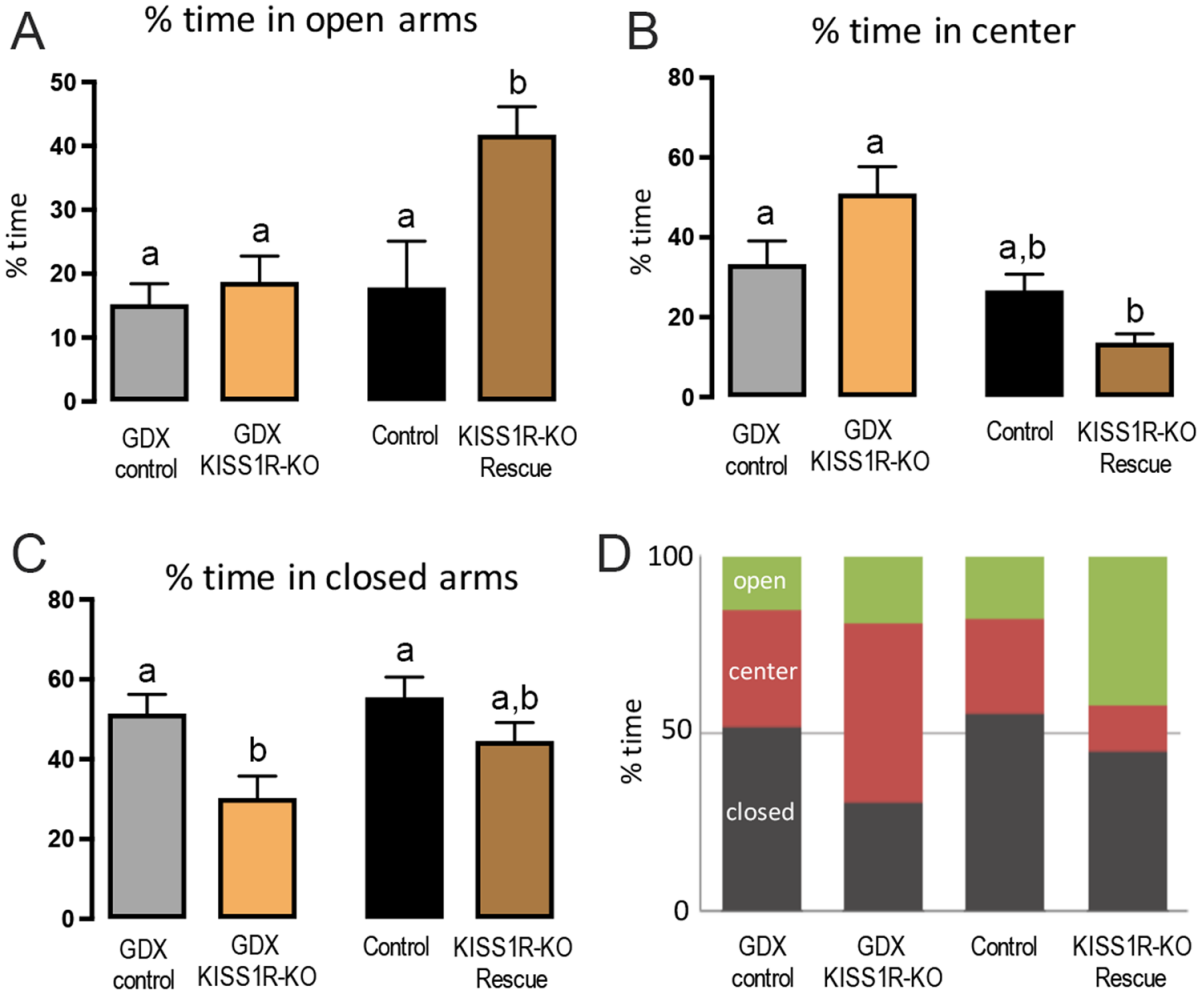
Elevated plus maze performance of gonadectomized (GDX) and intact KISS1R-KO mice. Different letters above histograms indicate statistically significant differences between GDX control (N = 9), GDX KISS1R-KO (N = 7), intact control (N = 8) and intact KISS1R-KO rescue (N = 9) mice. (**A**) Histograms showing the percentage of time mice spent in the open arms of the maze. b = *P* < 0.01, Tukey’s post-hoc multiple comparisons against all other groups. (**B**) Histograms showing the percentage of time mice spent in the center of the maze. a. vs b. = *P* < 0.05, Tukey’s post-hoc multiple comparisons against GDX groups. (**C**) Histograms showing the percentage of time mice spent in the closed arms. b = *P* < 0.05, Tukey’s post-hoc multiple comparisons against KISS1R-KO groups. (**D**) Summary providing a comparison of percentage of time spent in all three compartments for each group.

The number of times mice entered into the open arms during their 5 min in the EPM did not vary across the experimental groups; GDX control 7.0 ± 0.8, GDX KISS1R-KO 8.0 ± 0.8, intact control 7.1 ± 1.6, intact KISS1R-KO rescue 6.9 ± 0.8. However, the percentage of time spent in the different regions of the EPM was different for both GDX and intact experimental groups (Fig. 1A–C). The % of time spent in the open arms was significantly different across genotype (F(1,29) = 7.77, *P* = 0.009) and gonadal status (F(1,29) = 6.76, *P* = 0.015), with an interaction between the two (F(1,29) = 4.32, *P* = 0.046) and *post-hoc* tests showing that KISS1R-KO rescue mice spent approximately twice as much time in the open arms compared with controls (*P* = 0.008, Tukey’s post-hoc multiple comparisons) and all GDX mice (*P* < 0.01)(Fig. 1A). With regard to the % of time spent in the center of the EPM, a significant effect of gonadal status (F(1,29) = 20.47, *P* < 0.0001) and interaction between genotype and gonadal status (F(1,29) = 10.01, *P* = 0.003) was found. *Post-hoc* tests showed no differences between the two GDX groups or two intact groups but intact KISS1R-KO rescue mice spent significantly less time in the centre compared with both GDX groups (*P* < 0.05, Tukey’s post-hoc multiple comparisons)(Fig. 1B). For the % of time spent in the closed arms, a significant effect of genotype (F(1,29) = 10.39, *P* = 0.003) was identified with GDX KISS1R-KO mice spending less time in the closed arms compared with both GDX and intact controls (*P* < 0.05, Tukey’s post-hoc multiple comparisons)(Fig. 1C).

When compiled as percent event histograms for each group (Fig. 1D), the overall behaviour of GDX controls and intact controls appeared similar, whereas both KISS1R-KO mouse lines spent more time in the centre and open compartments of the EPM.

### Open Field Test (OFT)

**Figure 2:**
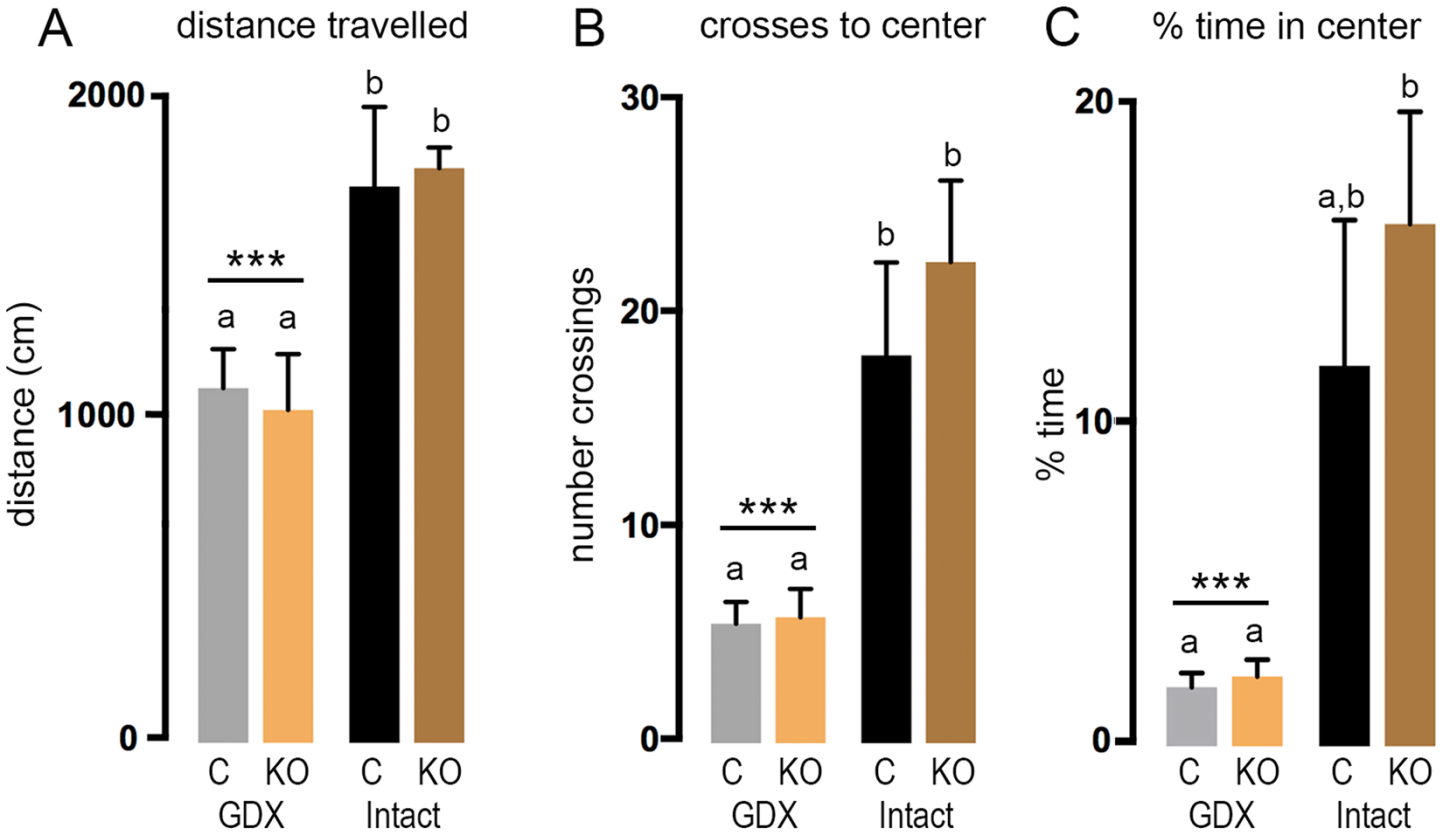
Open field trial performance of gonadectomized (GDX) and intact KISS1R-KO mice. Different letters above histograms indicate statistically significant differences between GDX control (GDX C; N = 9), GDX KISS1R-KO (GDX KO; N = 7), intact control (Intact C, N = 8) and intact KISS1R-KO rescue (Intact KO, N = 9) mice. Bars with *** above GDX group indicate significant difference between all GDX and all intact mice for each parameter (*P* < 0.001, 2-way ANOVA). (**A**) Histograms showing average total distance travelled over the 3 trials. b vs. a = *P* < 0.05, Tukey’s post-hoc multiple comparisons. (**B**) Histograms showing the average number of crossings into the inner quadrant (center) over the 3 trials. b vs. a = *P* < 0.001, Tukey’s post-hoc multiple comparisons. (**C**) Histograms showing the average percentage of time spent in the inner quadrant over the 3 trials. b vs. a = *P* < 0.01, Tukey’s post-hoc multiple comparisons.

The average distance travelled by mice across all three trials did not vary between control and KISS1R-KO mice in either intact or GDX groups (Fig. 2A). However, as a group, intact mice were found to travel significantly further (~60%) than GDX animals (effect of gonadal status, F(1,29) = 17.04, *P* = 0.0003) with both GDX groups travelling less than both intact groups (*P* < 0.05, Tukey’s post-hoc multiple comparisons). When examined across the three trials, intact and GDX mice were found to travel the same distance as each other on Day 1 (F(1,29) = 4.02, *P* = 0.054), with intact mice travelling further than GDX animals on Days 2 (F(1,29) = 15.43, *P* = 0.0005) and Day 3 (F(1,29) = 21.78, *P* < 0.0001)(data not shown).

The number of crossings to the center quadrant also showed no differences between genotype (F(1,29) = 0.603, *P* = 0.444), but over two-fold more crosses in intact mice compared with GDX animals (F(1,29) = 23.3, *P* < 0.0001)(Fig. 2B). Both GDX groups made fewer crossing to the center quadrant than both intact groups (*P* < 0.05, Tukey’s post-hoc multiple comparisons) (Fig. 2B).

The % of time spent in the inner quadrant exhibited the same relationship with no differences between genotype (F(1,29) = 0.679, *P* = 0.417), but intact mice spending over 5-times longer in the inner quadrant compared to GDX mice (F(1,29) = 17.38, *P* = 0.0003) (Fig. 2C). The same relationship between intact and GDX mice was observed on each of the three trial days (data not shown).

### Morris Water Maze (MWM)

**Figure 3:**
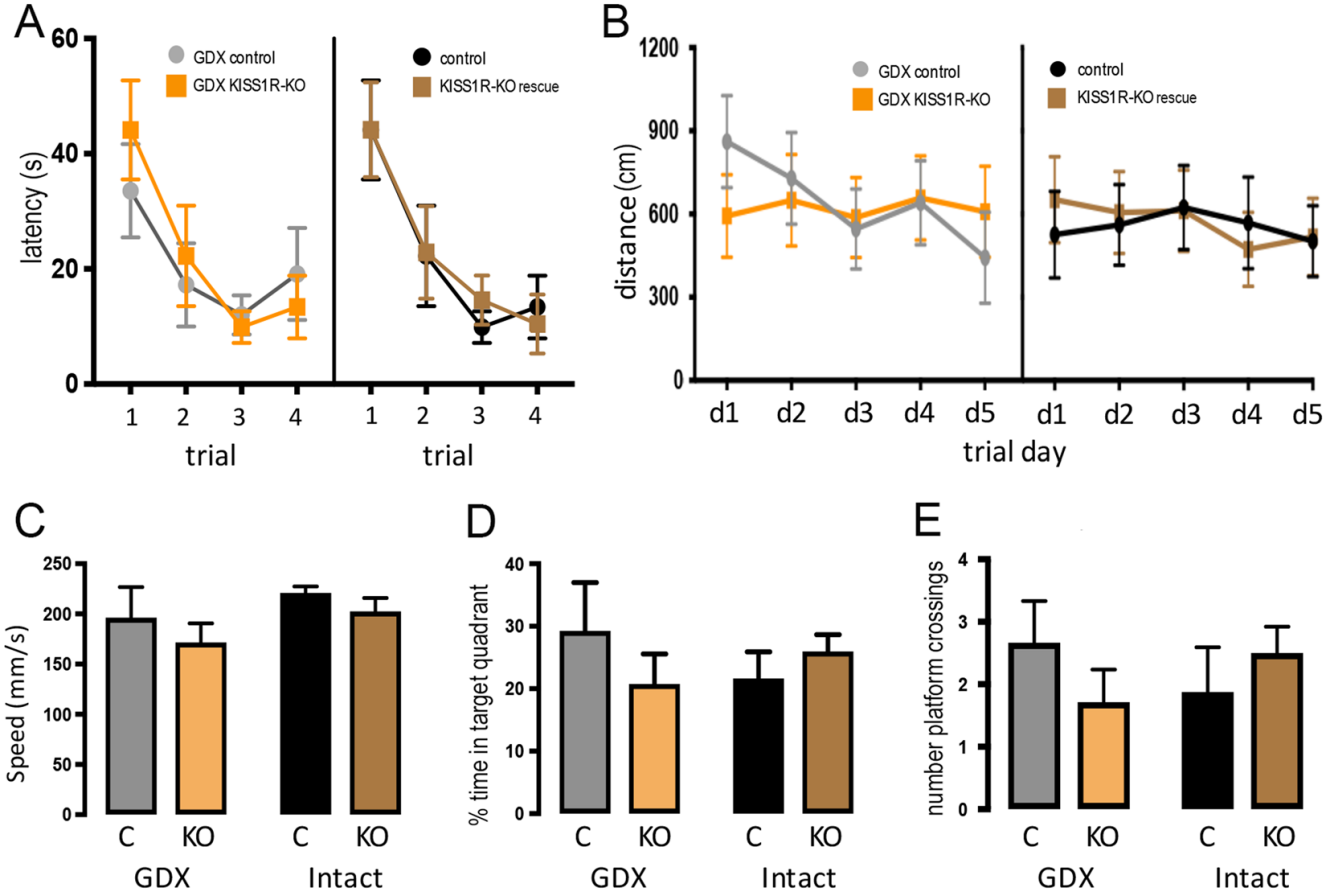
Morris water maze performance of GDX control (GDX C; N = 9), GDX KISS1R-KO (GDX KO; N = 7), intact control (Intact C, N = 8), and intact KISS1R-KO rescue (Intact KO, N = 9) mice. (**A**) Latency to reach a cued platform across 4 daily trails. GDX and intact groups are displayed side-by-side for clarity. No significant differences were detected between any of the 4 groups (*P* > 0.05; repeated-measures 2-way ANOVA). (**B**) Distance travelled to reach a hidden platform in the NE quadrant across the 5 trial days. GDX and intact groups are displayed side-by-side for clarity. No significant differences were detected between any of the 4 groups (*P* > 0.05; repeated-measures 2-way ANOVA). (**C**–**E**) Performance of mice in probe tests where the platform was removed. No significant differences were detected in swim speed, percentage of time spent in the target quadrant, or the number of times the mice crossed the prior location of the training platform (*P* > 0.05; 2-way ANOVA).

All mice were found to learn the cued task at an equivalent rate (Fig. 3A) with no significant differences between genotype or gonadal status (*P* > 0.05 for all comparisons, repeated-measures 2-way ANOVA). Performance in the reference memory spatial learning task was also not found to be different between any groups. Swimming velocity was the same and distance travelled (Fig. 3B) did not differ depending on genotype or gonadal status (*P* > 0.05). Probe tests also failed to reveal any significant differences in swimming speed (Fig. 3C), the percentage of time in the target quadrant (Fig. 3D) or number of crossings over the training platform location (Fig. 3E)(P > 0.05 for all comparisons, 2-way ANOVA).

## Discussion

We report here that KISS1R signaling in the male mouse is required for normal anxiety-like behavior in relation to the EPM. The full expression of this phenotype is dependent upon normal circulating levels of gonadal steroids. In addition, we find that gonadectomy has a potent anxiogenic effect on behavior in the OFT. No deficits in spatial learning were detected in relation to gonadectomy or the deletion of KISS1R signaling. Together, these observations suggest that kisspeptin and likely testosterone signaling can impact upon different neural circuitries mediating different aspects anxiety behavior in male mice.

### Effect of gonadectomy on anxiety-like behavior in male mice

Studies in male rats have reported contradictory results regarding the effects of castration upon anxiety-like behaviors in the EPM. Whereas an early study described increased anxiety following gonadectomy^30^, others have found no effect or even decreased anxiety^31–33^. Although less has been done with mice, a similar situation exists where investigators have reported gonadectomy to increase anxiety-like behaviors^34^ or have little effect^35,36^. Studies with testicular feminized rats and mice, that have life-long dysfunctional androgen receptor signaling, have demonstrated increased aspects of anxiety in both species^37,38^. One common observation with rats and mice, is that the effects of adult castration are often dissimilar in different behavioral tests of anxiety^33,34^. For example, Benice and Raber reported that GDX male mice travelled less distance and spent less time in the center of the OFT, but that gonadectomy had no impact upon time in the open arms of an elevated zero maze^34^.

Our observations regarding the effects of gonadectomy on anxiety-like behaviors are essentially the same as those of Benice and Raber^34^. We also find that gonadectomy reduces the total distance travelled and time in the centre of the OFT, but has no significant impact upon the time spent in the open arms of the EPM. Discrepancies between measures of anxiety in the OFT and EPM are relatively common^39,40^ and likely arise from behavioural tests measuring different facets or dimensions of anxiety. Thus, gonadectomy, and presumably testosterone, appears to selectively impact upon anxiety circuitry modulating the impetus for male mice to explore and remain exposed in the open. As noted previously^34,41^, there appears to be no impact of gonadectomy on cued or spatial learning in the reference memory water maze task.

### A role for KISS1R signaling in anxiety-like behavior

The major observation of this study is that intact KISS1R-KO rescue mice spend twice as much time in the open arms of the EPM compared to intact controls. Although a trend for reduced anxiety behavior was also evident in the OFT, this was not found to be statistically significant. This indicates that KISS1R-dependent signaling is normally facilitating the activity of anxiogenic neural circuits operative in the EPM. Interestingly, these circuits appear to be different to the anxiety networks addressed by testosterone as gonadectomy had a substantial impact upon performance in the OFT. The simplest interpretation of these observations would be that testosterone is involved in modulating anxiety circuits related to the fear of open spaces (OFT) whereas KISS1R signaling is important for anxiety related to the fear of heights (EPM). The possibility that these effects reflect strain differences seems unlikely as the C57BL/6 J and 129 strains perform very similarly in the EPM and OFT^42^. Compatible with the present results, a study in male rats has shown that intracerebroventricular administration of kisspeptin-13 causes anxiety in the EPM^43^.

We note that the anxiolytic phenotype of *Kiss1r*-null mice was only clearly observed in intact KISS1R-KO rescue mice. The GDX KISS1R-KO mice exhibited a similar but less robust phenomenon with less time spent in the closed arms and a trend to spend longer in the center and open arms combined. This may suggest that testosterone is involved in modulating KISS1R-dependent anxiety parameters in the EPM. More generally, this observation highlights the potential importance of normal levels of circulating gonadal steroids when interrogating kisspeptin-KISS1R signaling throughout the body.

The deletion of *Kiss1r* was found to have no effects upon performance in the Morris water maze. This indicates that there is no critical role for KISS1R signaling in brain circuits responsible for spatial learning and memory in male mice^44^. A recent study reported that infusion of kisspeptin-13 into the third ventricle ameliorated memory loss in amyloid beta-treated mice^45^. As this effect was inhibited by a GnRH receptor antagonist, it is possible that the memory changes in that study were indirect resulting from increases in circulating gonadal hormone levels secondary to kisspeptin activation of GnRH neurons.

A variety of limbic brain regions contribute to anxiety-related behaviors^21^ and it is conceivable that deleted KISS1R signaling in any of these areas could underlie the specific anxiolytic effects reported here in KISS1R-KO mice. However, of these various regions, it is only the granule cells of the hippocampal dentate gyrus that express *Kiss1r* at any appreciable level^18^. Recent hippocampal optogenetic studies have highlighted the importance of dorsal granule cells in the modulation of exploratory behavior and ventral granule cells in the regulation of anxiety^46^. As the EPM tests the conflict between exploration and fear of a potentially threatening environment, KISS1R signaling may be important in either or both the dorsal and ventral dentate. Although other ligands are possible for KISS1R^13^, it is notable that electrophysiological studies have shown that kisspeptin increases the excitability of granule cells in the dentate^20^. Thus, it is plausible that a potential reduction in granule cell excitability in KISS1R-KO mice may underlie their inclination to spend more time in the open arms of the EPM.

To our knowledge, the anxiety of the few humans identified with mutations in *KISS1R* has not been assessed. However, roles for kisspeptin outside the human hypothalamus have been suggested recently with the finding that the peripheral administration of kisspeptin can alter limbic brain activity, including the hippocampus, in relation to sexual and emotional stimuli^47^. Given our present observation of a specific anxiety deficit in *Kiss1r* -null male mice, it may be important to consider the possibility of effects of kisspeptin on anxiety when designing new therapies for treating reproductive disorders in the clinic.

## Materials and Methods

Adult male 129S6 global *Kiss1r*−/− (GDX KISS1R-KO) and littermate *Kiss1r*+/+ (GDX control) mice^7^ were GDX three weeks before beginning experiments when 4–6 months old. Adult male 129P2/C57BL/6 J *Kiss1r*^−/−^; Tg^+/−^ (KISS1R-KO rescue) and littermate Tg^+/−^ (control) mice^27^ were used for experiments at 4–6 months of age. Littermate null and control mice for each experiment were housed together three to four mice per cage under a 12 h light-dark cycle (lights on 19:00) at 20 ± 1 °C with 40 ± 5% humidity, and food and water available *ad libitum*. Experiments were undertaken during the animals’ dark phase under sodium lighting in the University of Otago Behavioural Phenotyping Unit. All experimentation was approved by the University of Otago Ethics Committee and undertaken in accordance with the relevant guidelines and regulations.

Mice (GDX control, N = 9; GDX KISS1R-KO, N = 7; control, N = 8; KISS1R-KO rescue, N = 9) were tested by an investigator blind to the experimental group and genotype. Mice were habituated to the investigator by daily handling for four days prior to behavioral testing. This consisted of one trial in the EPM (day 1), three trials in an OFT (days 1–3) and testing in a MWM (days 4–10). Mice were tracked and analysed using an overhead camera and the TopScan behavioural analysis system (Clever Sys Inc., Washington, USA).

### EPM

The maze consisted of four arms (35 cm long, 5 cm wide) extending out from a central square (5 × 5 cm) with two arms having 20 cm high walls. Mice were placed in the central square facing an open arm and their activity tracked for 5 min. The number of times a mouse entered fully (whole body) into an open or closed arm and the % of time a mouse spent in the closed, centre, and open arms of the maze were measured.

### OFT

Each mouse was tested in the OFT after completing the EPM on Day 1 and then tested again on Days 2 and 3. The open field was rectangular open box (40 cm × 40 cm with 20 cm high walls) with the mouse placed in the centre of the open field and its activity tracked for 10 min. The total distance travelled, the number of crossings into the middle portion of the arena (center quadrant, 24 × 24 cm), and percentage of time spent in the center quadrant were measured.

### MWM

The day after completing the OFT, mice were tested in a circular water maze 100 cm in diameter and 35 cm deep maintained at 22 °C. A rectangular platform (6 cm diameter) was placed 1 cm above or below the surface of the water in various locations within the pool. *Cued water maze training* (Day 4) was undertaken by placing a blue flag on the platform located above the water in the middle of the maze and placing mice with their nose to the wall of each of the four quadrants. Each trial lasted until the mouse had located the platform or until 60 s had elapsed at which time mouse was placed on the platform for 10 s. Two trials were undertaken in the morning and a further two trials in the afternoon. The time taken for mice to locate the platform on each trial was determined. *Spatial learning and memory* (Days 5–9) was tested over the next 5 days by locating the platform below the water and without a flag in the middle of the north-east (NE) quadrant. Mice were then placed in the pool in each of the quadrants as above and this was repeated on 5 consecutive days. The swimming velocity and distance required for mice to reach the platform was measured. Probe tests were undertaken on Days 7 and 9 before the spatial learning test by removing the platform from the pool and placing the mice with their nose to wall into the south-west quadrant. Mice were allowed to swim freely for 60 s and their swim velocity, number of crossing over the previous location of the platform, and % time spent in the NE quadrant measured.

Statistical Analysis: Comparisons across the four experimental groups were assessed with normal or repeated measures 2-way ANOVA with Tukey’s *post-hoc* multiple comparison tests where required. Statistical analysis was performed with GraphPad Prism 7 software.

### Data availability

The datasets generated during and/or analysed during the current study are available from the corresponding author on reasonable request.
